# Differentiation of Meningiomas and Gliomas by Amide Proton Transfer Imaging: A Preliminary Study of Brain Tumour Infiltration

**DOI:** 10.3389/fonc.2022.886968

**Published:** 2022-05-11

**Authors:** Han-Wen Zhang, Xiao-Lei Liu, Hong-Bo Zhang, Ying-Qi Li, Yu-li Wang, Yu-Ning Feng, Kan Deng, Yi Lei, Biao Huang, Fan Lin

**Affiliations:** ^1^ Department of Radiology, The First Affiliated Hospital of Shenzhen University, Health Science Center, Shenzhen Second People's Hospital, Shenzhen, China; ^2^ Department of Radiology, The Seventh Affiliated Hospital, Sun Yat-sen University, Shenzhen, China; ^3^ Department of Radiology, Songgang People's Hospital, Shenzhen, China; ^4^ Research Department, Philips Healthcare, Guangzhou, China; ^5^ Department of Radiology, Guangdong Provincial People's Hospital, Guangdong Academy of Medical Sciences, Guangdong, China

**Keywords:** amide proton transfer, differential diagnosis, glioma, meningioma, MRI, tumour invasion

## Abstract

**Background:**

Gliomas are more malignant and invasive than meningiomas.

**Objective:**

To distinguish meningiomas from low-grade/high-grade gliomas (LGGs/HGGs) using amide proton transfer imaging (APT) combined with conventional magnetic resonance imaging (MRI) and to explore the application of APT in evaluating brain tumour invasiveness.

**Materials and Methods:**

The imaging data of 50 brain tumors confirmed by pathology in patients who underwent APT scanning in our centre were retrospectively analysed. Of these tumors, 25 were meningiomas, 10 were LGGs, and 15 were HGGs. The extent of the tumour-induced range was measured on APT images, T2-weighted imaging (T2WI), and MRI enhancement; additionally, and the degree of enhancement was graded. Ratios (R^APT/T2^ and R^APT/E^) were obtained by dividing the range of changes observed by APT by the range of changes observed *via* T2WI and MR enhancement, respectively, and APT_mean_ values were measured. The Mann–Whitney U test was used to compare the above measured values with the pathological results obtained for gliomas and meningiomas, the Kruskal-Wallis test was used to compare LGGs, HGGs and meningiomas, and Dunn’s test was used for pairwise comparisons. In addition, receiver operating characteristic (ROC) curves were drawn.

**Results:**

The Mann–Whitney U test showed that APT_mean_ (*p*=0.005), R^APT/T2^ (*p*<0.001), and R^APT/E^ (*p*<0.001) values were statistically significant in the identification of meningioma and glioma. The Kruskal-Wallis test showed that the parameters APT_mean_, R^APT/T2^, R^APT/E^ and the degree of enhancement are statistically significant. Dunn’s test revealed that R^APT/T2^ (*p*=0.004) and R^APT/E^ (*p*=0.008) could be used for the identification of LGGs and meningiomas. APT_mean_ (*p*<0.001), R^APT/T2^ (*p*<0.001), and R^APT/E^ (*p*<0.001) could be used for the identification of HGGs and meningiomas. APT_mean_ (*p*<0.001) was statistically significant in the comparison of LGGs and HGGs. ROC curves showed that R^APT/T2^ (area under the curve (AUC)=0.947) and R^APT/E^ (AUC=0.919) could be used to distinguish gliomas from meningiomas.

**Conclusion:**

APT can be used for the differential diagnosis of meningioma and glioma, but APT_mean_ values can only be used for the differential diagnosis of HGGs and meningiomas or HGGs and LGGs. Gliomas exhibit more obvious changes than meningiomas in APT images of brain tissue; this outcome may be caused by brain infiltration.

## Advances in Knowledge

1. APT combined with conventional MR can be very good for the differential diagnosis of gliomas and meningiomas. APT can be used to differentiate among LGGs, HGGs and meningiomas.
2. Glioma APT images are larger than conventional MR images; therefore, gliomas are more obvious on these images than relatively benign meningiomas. This may be related to the infiltration of brain tumors.

## 1 Introduction

The Warburg effect, the theory that tumors metabolize acidic substances, is increasingly recognized by an increasing number of scientists studying tumors (1). In cutting-edge research, scientific experiments involving the abnormal accumulation of a series of acidic metabolites, such as methylglutaric acid, have repeatedly verified this viewpoint (2, 3). In the field of gliomas, Varun Venkataramani et al. proposed that glutamatergic synaptic input of glioma cells drives brain tumour progression, especially glioma progression (4). Compared with meningiomas with a significantly better survival prognosis, gliomas undoubtedly have a stronger ability to infiltrate the brain.

Due to the high recurrence rate and poor prognosis, gliomas have become a research hotspot in medicine (5). The main reason for the high recurrence rate of gliomas is the inability to effectively measure the boundary of the tumour infiltrated so that the gliomas cannot be radically removed (6). Neither long-term temozolomide chemotherapy nor radiotherapy in the later stage can effectively cure patients (7, 8). However, most meningiomas originating from the meninges, except for a very small number of malignant meningiomas, can be cured by excision. Meningiomas are directly supplied by meningeal blood vessels and show significant enhancement on enhanced magnetic resonance (MR) scans, and high-grade glioma (HGG)-induced blood–brain barrier (BBB) disruption can achieve a similar degree of enhancement (9).

According to the Warburg effect, malignant tumors cause acidic substances to accumulate in the infiltrating area. At present, clinical imaging technologies that can measure acidic metabolites include MR spectroscopy (MRS) and amide proton transfer imaging (APT) based on chemical exchange saturation transfer (CEST) (10, 11). APT technology can measure the amide proton content and indirectly reflect the protein content and pH value of a substance (12). At present, research on APT in gliomas mainly focuses on the use of the APT value for the evaluation of preoperative tumour malignancy and the identification of postoperative recurrence and pseudo-progression (13–15). However, there are only two studies on meningiomas, one on the content of Ki-67 in meningiomas and the other on the differential diagnosis of typical and atypical meningiomas (16, 17). At present, there is no research on the differential diagnosis of gliomas and meningiomas using APT technology.

In this study, APT was used to evaluate gliomas and meningiomas to obtain APT values, and the range of APT changes caused by tumors was measured and compared with the range of changes observed by conventional imaging of tumors. These analyses were performed to explore whether the changes in APT values and ranges caused by meningiomas and gliomas can be used for differential diagnosis.

## 2 Materials and Methods

### 2.1 Patients

From January 2021 to February 2022, one hundred brain tumour patients who underwent Philips Ingenia 3.0 Tesla (T) MR imaging (MRI) scans were collected in our centre. Inclusion criteria were as follows (1): complete clinical data, no history of trauma and radiotherapy and chemotherapy (2); complete routine imaging before surgery, including T1-weighted imaging (T1WI), T2-weighted imaging (T2WI), enhanced scan, diffusion-weighted imaging (DWI) and APT, with satisfactory image quality diagnosis requirements; and (3) patients with postoperative pathological diagnosis of meningioma or glioma after surgical treatment.

Finally, 50 patients (25 males and 25 females) were included, including 25 patients with meningioma (12 males and 13 females) and 25 patients with gliomas (13 males and 12 females), aged 16-71 (48 ± 13) years old. This retrospective study was approved by the regional ethics committee and exempted from informed consent.

### 2.2 MRI

We scanned patients with a Philips Healthcare 3.0T Ingenia MR scanner (Philips Healthcare, Amsterdam, The Netherlands) using a 20-channel head coil. Patients fasted for 4-6 h before scanning. The localization line was located at the inferior border of the corpus callosum, and the scanning range was the whole brain. T1WI, T2WI, T2W-fluid attenuated inversion recovery (FLAIR), DWI, and APT scans were performed first, and then enhanced scans were performed.

#### 2.2.1 Conventional MR Sequence

1. Axial T1WI parameters were as follows: repetition time (TR)/echo time (TE) = 2034/20 milliseconds (ms), section thickness = 5 mm, field of vision (FOV) = 230×210 mm, and matrix = 328×258. 2. Axial T2WI parameters were as follows: TR/TE = 3000/105 ms, section thickness = 5 mm, FOV = 230×210 mm, and matrix = 328×258. 3. Coronal T2W-FLAIR parameters were as follows: TR/TE = 9000/81 ms, section thickness = 5 mm, inversion time = 2500 ms, FOV = 230 × 210 mm, and matrix = 328 × 258). 4. Axial DWI parameters were as follows: the B value of DWI is B = 0 and B = 1000 s/mm2, TR/TE = 3741/80 ms, section thickness = 5 mm, FOV = 230×210 mm, FOV = phase 100%, and matrix = 128 ×97.

#### 2.2.2 APT and T1W-Enhanced Sequence

1. APT images were acquired axially (TR/TE=6306/8.3 ms, section thickness=5 mm, FOV=230×180 mm, and matrix=128×100).
2. An intravenous injection of gadodiamide (Omniscan, GE Healthcare, Dublin, Ireland) was carried out at an injection rate of 3.5 mL/s *via* a power injector (0.1 mmol/kg), followed by flushing with 10 mL of normal saline. Sagittal T1WI-enhanced parameters were as follows (TR/TE=6.8/3.1 ms, section thickness=1 mm, FOV=240×240 mm, and matrix=240×240).

### 2.3 Pathological Results

The pathological data of all patients included intraoperative pathology and immunohistochemical results. The pathological results of all glioma patients with complete molecular sequencing included isocitrate dehydrogenase, O6-methylguanine-DNA methyltransferase, telomere reverse transcriptase, etc.

The final pathological findings of the patients were obtained according to the 2016 edition of the CNS Tumour Classification.

### 2.4 Image Analysis

Conventional MR images were used for tumour extent measurements using ITK-SNAP-3.8.0 (University of Pennsylvania, Philadelphia, PA). The localization of the solid area of the tumour was performed on the T1WI structural image by three senior physicians, and the T2WI and enhanced scans were delineated. T2WI scans were marked, and the enhancement range was denoted as R^T2^ and R^E^. A region of interest (ROI) was selected to avoid surrounding blood vessels, tumour necrosis and regions of cystic degeneration. At the same time, for the enhanced scan images, we scored the degree of enhancement on a 1-4 scale (1-no enhancement, 2-slight enhancement, 3-moderate enhancement, and 4-significant enhancement).

APT mean (APT_mean_) and range measurements (R^APT^) were performed on APT images of tumors using IntelliSpace Portal 10.0 (Philips Medical, The Netherlands). T1WI structural images and the contralateral normal tissue areas served as references for the delineation of the range of APT image changes caused by tumors.

Finally, we compared R^APT^ with R^T2^ and R^E^ to obtain the corresponding ratios (R^APT/E^ and R^APT/T2^) to determine whether the range of APT increased.

### 2.5 Statistical Analysis

We used the Statistical Package for the Social Sciences (SPSS v.19, Chicago, IL, USA) for data statistics. The intra/interclass correlation coefficients (ICCs) were used to calculate consistency between reviewers. Two-sided *p* values<0.05 were considered statistically significant. All data were calculated using the Shapiro-Wilk test, and the result was *p*<0.05, indicating that the data did not conform to a normal distribution. Because the data did not follow a normal distribution, the Mann–Whitney U test was used to evaluate differences in enhancement and APT images based on APTmean, R^APT/T2^, and R^APT/E^ between gliomas and meningiomas. The Kruskal-Wallis test was used to compare LGGs, HGGs and meningiomas, and Dunn’s test was used for pairwise comparisons. The area under the curve (AUC), confidence interval (CI) and cut-off values of the different types were calculated by receiver operating characteristic (ROC) curve analysis and the Youden Index. Following Bonferroni correction, *p*<0.01 indicated statistical significance. All statistical graphs were made using GraphPad Prism 7 (Prism 7, La Jolla, California).

## 3 Results

### 3.1 Patients

The age and sex of the patients and the range of imaging (R^T2^, R^E^, and R^APT^) were not statistically significant. The interobserver agreement of all measurements between the three reviewers was excellent, with ICC values greater than 0.80. Basic patient information and imaging information are shown in **Table 1**.

**Table 1:** Clinical characteristics, tumour range of MR imaging and parameters related to APT based on differences in patients with glioma and meningioma.

Variable	Glioma (n=25)	Meningioma (n=25)	*p*
Clinical characteristics			
Age, years	48.68 ± 12.77	47.44 ± 15.08	0.793
Sex			0.779
Male	13 (52%)	12 (48%)	
Female	12 (48%)	13 (52%)	
MR information			
Degree of enhancement	2.84 ± 0.94	3.48 ± 0.51	0.011
Tumor range of MRI (mm^2^)			
R^T2^	1394.00 ± 1097.50	1720.00 ± 1699.00	0.831
R^E^	1558.00 ± 1083.50	1764.00 ± 1678.00	0.992
R^APT^	2175.00 ± 1219.00	1764.00 ± 1723.00	0.200
Parameters related to APT			
APT_mean_	4.28 ± 2.43	2.52 ± 0.63	0.005
R^APT/T2^	120.5% ± 23.0%	103.5% ± 6.0%	<0.001
R^APT/E^	127.0% ± 32.0%	102.4% ± 6.0%	<0.001

Ps: p<0.05 indicates statistical significance. Values are the median ± Interquartile Range (IQR).

*Degree of enhancement does not represent a specific value. Meningiomas are generally moderately or significantly enhanced, while gliomas can have various degrees of enhancement; LGGs are generally slightly or moderately enhanced, and HGGs are generally moderately or significantly enhanced.

(1-no enhancement, 2-slight enhancement, 3-moderate enhancement, 4-significant enhancement).

### 3.2 Differentiation of Meningiomas and Gliomas by APT

Mann–Whitney U test results showed that the degree of enhancement (*p*=0.011), APT_mean_ values (*p*=0.005), R^APT/E^ values (*p*<0.001) and R^APT/T2^ values (*p*<0.001) were statistically significant. This shows that R^APT/E^ and R^APT/T2^ can be used to distinguish gliomas and meningiomas to a better degree, and the change in APT range induced by gliomas is more obvious than that of meningiomas. Thus, the APT_mean_ and enhancement degree can help differentiate gliomas from meningiomas.

**Figure 1:**
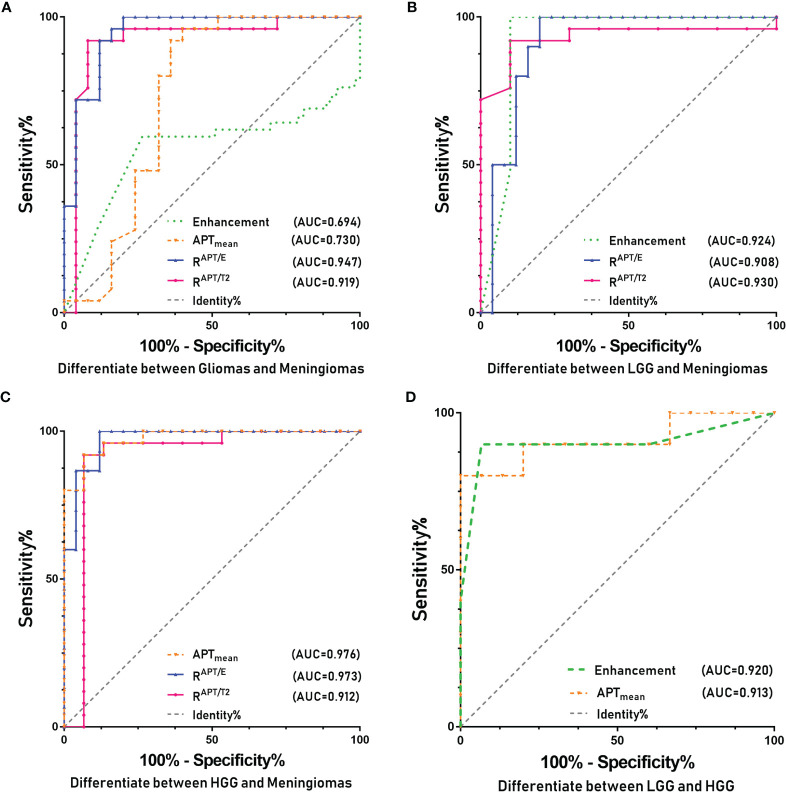
ROC curves of the various parameters of APT and for identifying different types of tumors. **(A)** Glioma and meningioma. **(B)** LGG and meningioma. **(C)** HGG and meningioma. **(D)** LGG and HGG.

The ROC curve analysis (**Figure 1A**) demonstrated that the R^APT/E^ (AUC=0.947, CI: 0.886-1.000) and R^APT/T2^ (AUC=0.919, CI: 0.886-1.000) values were significantly different between patients in the glioma and meningioma groups (*p*<0.001), which allowed a high diagnostic efficiency. The cut-off value for R^APT/E^ was 1.116, with a sensitivity of 92% and a specificity of 92%. The cut-off value of R^APT/T2^ was 1.092, with a sensitivity of 92% and a specificity of 88%. This shows that the changes in the APT range of most gliomas increased by more than 11.6% in contrast-enhanced images, and this change reached more than 9.2% when compared with that of T2WI images.

### 3.3 Differentiation of Meningiomas, LGGs and HGGs by APT

The Kruskal-Wallis test showed that the parameters APT_mean_, R^APT/T2^, R^APT/E^ and the degree of enhancement were statistically significant (*p*<0.001).

#### 3.3.1 Differentiation of Meningiomas From LGGs by APT

**Figure 2:**
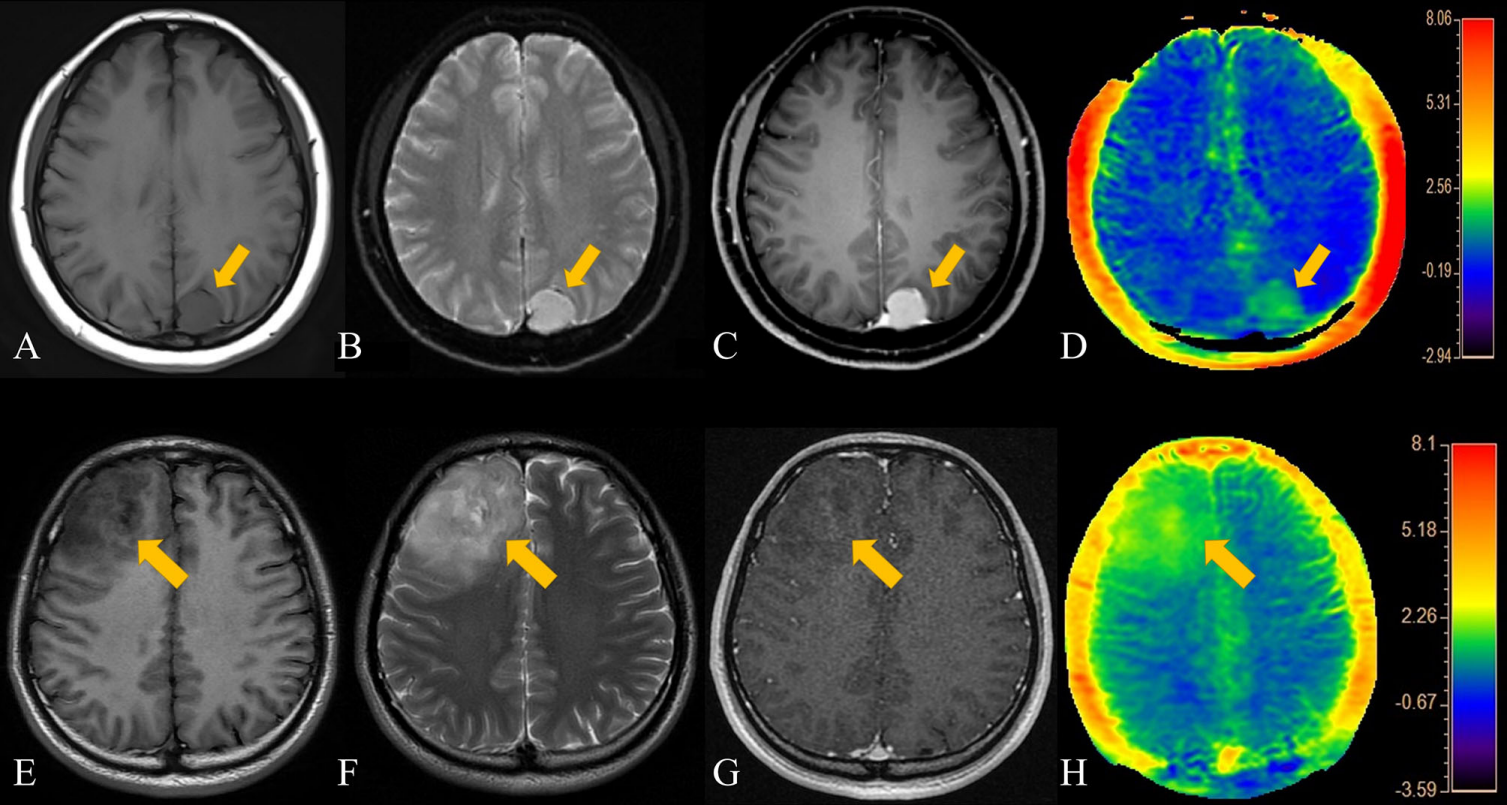
Patient 1, a 44-year-old female, presented with a left parietal lobe meningioma. The patient's T2WI, contrast-enhanced MRI, and APT range were similar. **(A)** T1-weighted imaging showed an iso-slightly low signal. **(B)** T2-weighted imaging showed an iso-slightly high signal. **(C)** Enhanced MR imaging showed marked enhancement. **(D)** APT imaging shows a slightly higher rate of amide proton transfer. Patient 2, a 34-year- old male, had an LGG in the right frontal lobe. The APT signal range of the patient was slightly larger than that on both the T2WI and enhanced scans. **(E)** T1-weighted imaging showed an iso-slightly low signal. **(F)** T2-weighted imaging showed an iso-slightly high signal. **(G)** Enhanced MRI showed mild enhancement. **(H)** APT imaging showed that the amide proton transfer rate slightly increased.

Dunn’s test results showed that the degree of enhancement (*p*<0.001), R^APT/E^ values (*p*=0.008) and R^APT/T2^ values (*p*=0.004) were statistically significant, but not APT_mean_ (*p*>0.01). Hence, the degree of enhancement, R^APT/E^ and R^APT/T2^ can be used to distinguish LGGs from meningiomas. The enhancement degree of meningiomas is obviously stronger than that of LGGs, and the changes in APT images induced by LGGs are more obvious than those of meningiomas. However, since the APT_mean_ of LGGs is close to that of meningiomas, this value cannot be used for differential diagnosis. **Figure 2** shows a comparison of APT and conventional MR images of LGGs and meningiomas.

The ROC curve analysis (**Figure 1B**) demonstrated that the R^APT/E^ (AUC=0.908, CI: 0.809-1.000), R^APT/T2^ (AUC=0.930, CI: 0.840-1.000), degree of enhancement (AUC=0.924, CI: 0.779-1.000) were significantly different between patients in the LGG and meningioma groups (*p*<0.01), which allowed high diagnostic efficiency. The cut-off value for R^APT/E^ was 1.104, with a sensitivity of 90% and a specificity of 86%. The cut-off value of R^APT/T2^ was 1.095, with a sensitivity of 90% and a specificity of 92%. This shows that the changes in the APT range of most LGGs increased by more than 10.4% on contrast-enhanced images, and this change also reached more than 9.5% when compared with that of T2WI images. The enhanced image had a cut-off value of 1.5, a sensitivity of 100% and a specificity of 80%. This indicates that the enhancement of meningiomas is significantly stronger than that of LGGs.

#### 3.3.2 Differentiation of Meningiomas From HGGs by APT

Dunn’s test results showed that the APT_mean_ (*p*<0.001), R^APT/E^ (*p*<0.001) and R^APT/T2^ (*p*<0.001) values were statistically significant. However, the degree of enhancement was not statistically significant (*p*>0.01). This shows that APT_mean_, R^APT/E^ and R^APT/T2^ can be used to distinguish HGG and meningioma to a better degree. The APT_mean_ of HGG is significantly higher than that of meningioma, and the changes in the APT image range brought by HGG are more obvious than those of meningioma. The enhancement degree of meningioma and HGG is mostly moderate or marked enhancement, so the degree of enhancement cannot be used to distinguish these two diseases.

In the ROC curve analysis (**Figure 1C**), R^APT/E^ (AUC=0.973, CI: 0.932-1.000), R^APT/T2^ (AUC=0.912, CI: 0.782-1.000), APT_mean_ (AUC=0.976, CI: 0.938-1.000) values were significantly different between patients in the HGG and meningioma groups (*p*<0.001), which allowed a high diagnostic efficiency. The cut-off value for R^APT/E^ was 1.123 with a sensitivity of 93.3% and a specificity of 88.0%. The cut-off value of R^APT/T2^ was 1.101, the sensitivity was 86.7%, and the specificity was 92.0%. This shows that compared to that on enhanced images, the change in the APT range of HGGs increased by more than 12.3%, and this change also reached more than 10.1% compared with that on T2WI scans. The cut-off of the APT value was 3.840, the sensitivity was 93.3%, and the specificity was 92.0%. This finding indicated that the APT_mean_ of HGGs was significantly higher than that of meningiomas.

#### 3.3.3 Differential Diagnosis of LGGs and HGGs by APT

Dunn’s test results showed that the degree of enhancement (*p*=0.001) and APT_mean_ *(p*<0.001) were statistically significant. However, R^APT/E^ (*p*=0.299) and R^APT/T2^(*p*=0.969) were not statistically significant (*p*>0.01). This shows that the APT_mean_ and the enhancement degree can better distinguish HGGs and LGGs. HGGs usually have a high degree of enhancement, and the APTmean is also higher than that of LGGs.

The ROC curve analysis (**Figure 1D**) demonstrated that the enhancement (AUC=0.920, CI: 0.768-1.000) and APT_mean_ (AUC=0.913, CI: 0.720-1.000) were significantly different between patients in the HGG and LGG groups (*p ≤* 0.001), which allowed a high diagnostic efficiency. The cut-off value of the APT value was 3.520, the sensitivity was 93.3%, and the specificity was 80.0%. The cut-off value for the degree of enhancement was 1.5, with a sensitivity of 100% and a specificity of 80%. This shows that the APT_mean_ of HGGs is higher, and the enhancement degree is above moderate enhancement.

**Figure 3:**
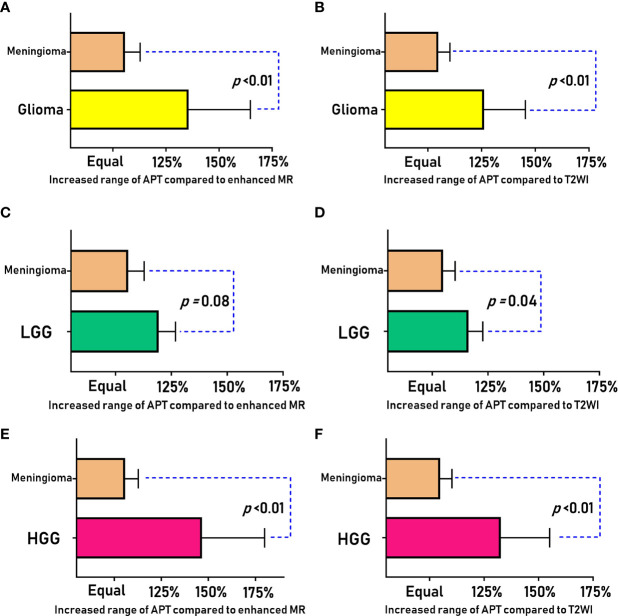
Comparison of the APT enlargement range (R^APT/T2^ and R^APT/E^) between meningiomas and gliomas (LGG and HGG).

We briefly summarize the above statistical results in **Table 2** for the differential diagnosis of HGG, LGG and meningioma. We sorted the invasiveness of glioma, LGG, and HGG compared with meningioma, as shown in **Figure 3**.

**Table 2:** APT and enhancement features to differentiate LGGs, HGGs, and meningiomas.

	LGGs and meningiomas	HGGs and meningiomas	LGGs and HGGs
Degree of enhancement	√, Enhancement degree of meningiomas is higher	X, Both were mainly significant enhancement	√, Enhancement degree of HGG is higher
APT_mean_	X, Both have relatively low APT_mean_	√, The APT_mean_ of HGG is significantly higher	√, HGG is significantly higher than LGG
R^APT/T2^	√, LGG changes are more pronounced	√, HGG changes are more pronounced	X, Both LGGs and HGGs have strong tumour invasiveness to the brain
R^APT/E^	√, LGG changes are more pronounced	√, HGG changes are more pronounced	X, Both LGGs and HGGs have strong tumour invasiveness to the brain

Ps: √-statistically significant, X-not statistically significant. (p<0.01 indicates statistical significance).

## 4 Discussion

APT technology was first established by Zhou et al. of Johns Hopkins University by applying the most classic two-pool model, namely, a free water pool (solution) and an amide proton pool (solute) (18). Since peptides and proteins in the body generally contain amide bonds (-NH), the resonance frequency of hydrogen protons is 0 ppm, the resonance frequency of amide protons is 3.5 ppm, and the asymmetrical magnetization transfer rate (MTRasym) calculated by using the Z-spectrum can reflect the APT rate (19). Amide protons were saturated under continuous selective saturating radio frequency (RF) pulse irradiation. Exchange with free water *via* chemical shifts resulted in a reduction in the water signal. The reduction in this signal can indirectly reflect the changes in metabolites and pH changes in the substance; hence, it can be used to determine the content of amide protons in this region (20). This study used APT technology to study patients with gliomas and meningiomas and found an interesting phenomenon. The changes in the range of APT images caused by gliomas were more obvious than those of relatively benign meningiomas.

The use of conventional MRI for the diagnosis of glioma and meningioma is quite mature. For example, typical meningiomas have some obvious signs, such as the “meningeal tail “ sign, which can be used for differential diagnosis. However, for the diagnosis of atypical meningioma or meningeal enhancement caused by glioma, APT technology can assist in the differential diagnosis to a certain extent. At the same time, combining APT with conventional MRI images has been shown to yield better diagnostic performance. For example, compared with significantly enhanced meningiomas, HGG has a higher APT_mean_ value, and the change range of APT images is larger than that of conventional images. APT measurements can help differentiate gliomas from meningiomas; however, these measurements are less effective for differentiating LGGs with a lower MTRasym from meningiomas. However, the enlargement range of APT compared with traditional MR images caused by HGGs or LGGs is more obvious than that of meningiomas. We cannot directly conclude that this is the extent of glioma brain infiltration. Moreover, we have no pathological confirmation, but this change does exist compared to more benign meningiomas. Our study can only indicate that APT imaging may have some value in the determination of malignant brain tumour boundaries, but there is no doubt that this approach can be used in the differential diagnosis between gliomas and meningiomas.

In fact, the use of APT in the differential diagnosis of brain tumors is well established, but its use has mainly focused on the differentiation of solitary brain metastases (SBMs) and glioblastomas (GBMs) (21). Both Yu and Kamimura’s studies found that the APT value of GBMs was higher than that of SBMs, which can be used for the differential diagnosis of GBMs (22, 23). However, it is interesting that, in Kamimura’s study, we found that the APT value in the nonenhanced T2WI-increased area around GBMs was significantly higher than that in the corresponding T2WI-increased area of SBMs. Our study on gliomas and meningiomas also had similar results. Relatively benign meningiomas had lower APT values, but we found that gliomas caused changes in the APT range that were even larger than the range detected by T2WI, and LGGs also had similar performance to HGGs. The range of these changes was more than 9%, and these changes can even be directly judged by the naked eye. Combined with the enhancement degree and APT value, these changes can effectively help clinicians accurately identify gliomas and meningiomas.

**Figure 4:**
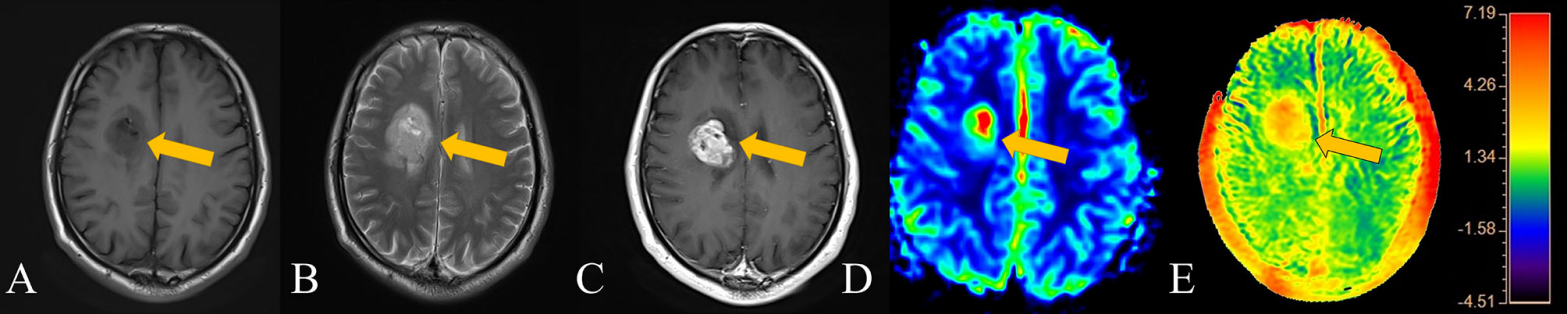
Patient 3, a 62-year-old male, has an HGG in the right basal ganglia. The APT signal range of the patient was obviously larger than that on both T2WI and enhanced scans and larger than that on PWI. **(A)** T1-weighted imaging showed an iso-slightly low signal. **(B)** T2-weighted imaging showed an iso-slightly high signal. **(C)** Enhanced MR imaging showed obvious enhancement. **(D)** PWI showed marked hyper-perfusion. **(E)** APT imaging shows that the amide proton transfer rate obviously increased.

“Ischaemic penumbra” is a concept of acute cerebral ischaemia, referring to the area of brain tissue that can be recovered with prompt medical treatment, usually referring to the area of brain tissue between DWI and perfusion-weighted imaging (PWI) (24). Gliomas also lead to the appearance of similar areas of tissue due to tumour brain infiltration, that is, the areas shown by conventional MR and the true borders of gliomas. The delineation of this boundary determines the extent of the operation and the prognosis of the patient’s survival (25). Similar to the use of intraoperative fluorescent probes, tumors can be radically removed as much as possible while preserving the patient’s normal brain tissue area (26). Our thinking is similar to that of most scholars; the changes in gliomas should first be the infiltration of microscopic tumour cells and the accumulation of metabolites and then the formation of tumors and corresponding tumor blood vessels (27). Notably, there are many techniques used in the preoperative evaluation of brain stromal tumors, including PWI, DWI, MRS, etc. Compared with the improved enhancement caused by the destruction of the BBB (usually HGGs are more likely to cause BBB disruption than LGGs), PWI can more effectively respond to the blood supply of gliomas, and the range of APT that can respond to substance metabolism should be larger than that of PWI (28, 29). Some of our patients also underwent PWI. For example, the suitable range for PWI in the patient with HGG in **Figure 4** was also significantly smaller than that of APT. If enough similar patients are evaluated, this result should be verifiable. Compared with PWI, DWI or conventional MRI, APT may have higher sensitivity in the early stage of brain tumour infiltration, and it also has a certain role in determining the tumour infiltration boundary. Thus, it is helpful for determining the scope of surgery.

Importantly, there are two main ways to verify the imaging boundary of glioma brain infiltration: 1. Pathological examination of the area showing changes on images; if the corresponding pathological results could be obtained, they could be directly confirmed. However, the main difficulty of this method lies in the extended clinical resection and the correspondence between pathological tissue and imaging. 2. For the correspondence between the scope of resection and preoperative images, the patients were followed up for recurrence, and the survival time was recorded. At present, many studies have proven that APT can be applied to the judgement of recurrence and pseudo-progression. However, if the lesions can be completely removed before surgery, the patients’ prognosis and treatment can be improved, and the need for a second surgery can be reduced (30).

Limitations of our research: 1. The number of patients was relatively small. 2. There was a lack of pathological results for whether the enlarged area of APT compared with conventional MR was a brain area infiltrated with tumors. 3. At present, this was only a study comparing APT and conventional MR images; if adequate functional imaging evaluation can be added, it will be possible to evaluate patients with brain tumors more comprehensively.

In conclusion, this study found that R^APT/E^, R^APT/T2^ and APT_mean_ can be better for the differential diagnosis of glioma and meningioma. APT combined with conventional imaging enhancement can better differentiate between LGGs, HGGs and meningiomas.

## Data Availability Statement

The raw data supporting the conclusions of this article will be made available by the authors, without undue reservation.

## Ethics Statement

The studies involving human participants were reviewed and approved by Clinical Research Ethics Committee of Shenzhen Second People’s Hospital. Written informed consent from the participants’ legal guardian/next of kin was not required to participate in this study in accordance with the national legislation and the institutional requirements.

## Author Contributions

H-WZ contributed to the conception of the study; H-BZ, Y-QL, and X-LL contributed significantly to analysis and manuscript preparation; KD, FL, and Y-LW performed the data analyses and wrote the manuscript; YL, BH, and Y-NF helped perform the analysis with constructive discussions. All authors contributed to the article and approved the submitted version.

## Funding

This study is supported by a grant from National Natural Science Foundation of China (Grant Number: 82071871),Youth Exploration Fund of Shenzhen Health Economics Society (Grant Number: 202211), Clinical Research Project of Shenzhen Second People's Hospital, China (Grant Number: 20193357021), and Clinical Research Project of Shenzhen Second People's Hospital, China (Grant Number: 20203357036).

## Conflict of Interest

The authors declare that the research was conducted in the absence of any commercial or financial relationships that could be construed as a potential conflict of interest.

## Publisher’s Note

All claims expressed in this article are solely those of the authors and do not necessarily represent those of their affiliated organizations, or those of the publisher, the editors and the reviewers. Any product that may be evaluated in this article, or claim that may be made by its manufacturer, is not guaranteed or endorsed by the publisher.
